# *Neisseria gonorrhoeae* Exposed to Sublethal Levels of Hydrogen Peroxide Mounts a Complex Transcriptional Response

**DOI:** 10.1128/mSystems.00156-18

**Published:** 2018-10-02

**Authors:** Sarah J. Quillin, Adam J. Hockenberry, Michael C. Jewett, H Steven Seifert

**Affiliations:** a Department of Microbiology-Immunology, Northwestern University Feinberg School of Medicine, Chicago, Illinois, USA; b Department of Chemical and Biological Engineering, Northwestern University, Evanston, Illinois, USA; c Center for Synthetic Biology, Northwestern University, Evanston, Illinois, USA; d Interdisciplinary Program in Biological Sciences, Northwestern University, Evanston, Illinois, USA; University of California, Irvine

**Keywords:** *Neisseria gonorrhoeae*, bleach, gonorrhea, hydrogen peroxide, neutrophils, superoxide, transcriptional regulation

## Abstract

Neisseria gonorrhoeae mounts a substantial transcriptional program in response to hydrogen peroxide (HP), a prominent reactive oxygen species (ROS) encountered during infection. We tested which strain FA1090 genes show differential transcript abundance in response to sublethal amounts of HP to differentiate HP-responsive signaling from widespread cellular death and dysregulation. RNA sequencing (RNA-Seq) revealed that 150 genes were significantly upregulated and 143 genes downregulated following HP exposure. We annotated HP-responsive operons and all transcriptional start sites (TSSs) and identified which TSSs responded to HP treatment. We compared the HP responses and other previously reported genes and found only partial overlapping of other regulatory networks, indicating that the response to HP involves multiple biological functions. Using a representative subset of responsive genes, we validated the RNA-Seq results and found that the HP transcriptome was similar to that of sublethal organic peroxide. None of the genes in the representative subset, however, responded to sublethal levels of HOCl or O_2_^-^. These results support the idea that N. gonorrhoeae may use variations in HP levels as a signal for different stages of infection.

**IMPORTANCE** The strict human pathogen Neisseria gonorrhoeae is the only causative agent of the sexually transmitted disease gonorrhea. This bacterium encounters hydrogen peroxide produced from host cells during infection, but the organism survives in the presence of this antimicrobial agent. This work shows that the bacterium responds to hydrogen peroxide by regulating the expression of many genes involved in multiple processes.

## Abstract (precis)

The strict human pathogen Neisseria gonorrhoeae is the only causative agent of the sexually transmitted disease gonorrhea. This bacterium encounters hydrogen peroxide produced from host cells during infection, but the organism survives in the presence of this antimicrobial agent. This work shows that the bacterium responds to hydrogen peroxide by regulating the expression of many genes involved in multiple processes.

## INTRODUCTION

While most members of the genus *Neisseria* are human and animal commensals, Neisseria gonorrhoeae and Neisseria meningitidis can elicit morbidity and mortality and are therefore considered pathogens. N. gonorrhoeae is a Gram-negative obligate human colonizer that resides primarily within the male and female genital tracts but can also colonize the rectal and ocular mucosa (1, 2). N. gonorrhoeae normally colonizes the genital mucosa, and many infected individuals are asymptomatic (3, 4). The primary clinical manifestations of gonorrhea are an influx of polymorphonuclear leukocytes (PMNs) to the site of infection and a resultant purulent exudate (5). PMNs have a number of antimicrobial defenses, including the ability to mount an oxidative burst, releasing damaging redox-active compounds called reactive oxygen species, or ROS. PMNs also have nonoxidative means of bacterial killing such as that by antimicrobial peptides, metal sequestration, and lytic enzymes (6, 7). The interactions between N. gonorrhoeae and PMNs are important for N. gonorrhoeae colonization, transmission, and replication and greatly contribute to the outcome of N. gonorrhoeae pathogenesis.

The underlying mechanisms mediating the interaction of N. gonorrhoeae with PMNs and the PMN oxidative burst are complex and not fully understood. Bacterial Opa proteins are important in the interaction between N. gonorrhoeae and PMNs. N. gonorrhoeae strains independently switch on and off expression of 11 to 13 *opa* genes (8, 9), and most Opa proteins interact with cellular receptors to mediate adherence to host cells and tissues (10, 11). Nongrowing or dead N. gonorrhoeae or OpaB/D-expressing N. gonorrhoeae bacteria induce an oxidative response (12, 13). The OpaB or OpaD variants of strain FA1090 bind to the CAECAM3 receptor on PMNs, eliciting a strong bactericidal oxidative burst that can effectively kill phagocytosed N. gonorrhoeae (11, 14). Live, growing, Opa-less N. gonorrhoeae bacteria do not induce an oxidative burst and are capable of actively suppressing the oxidative burst induced by nongrowing N. gonorrhoeae, Opa-expressing N. gonorrhoeae, or other microbial factors (12, 13, 15). Despite the ability of the PMNs to mount an oxidative burst, oxidative killing by PMNs appears to have only a minor role during infection, with most killing occurring by nonoxidative means (5, 6, 16, 17), except when certain Opa variants are expressed (11, 14).

N. gonorrhoeae has evolved multiple mechanisms to allow survival among PMNs (6, 18–23). Despite what appears to be a minor role for the oxidative burst in killing N. gonorrhoeae during infection, N. gonorrhoeae mounts a substantial transcriptional response to hydrogen peroxide (HP) (24). ROS, like HP, are known to react intracellularly to form free radicals, causing widespread damage to proteins, membranes, enzyme centers, and DNA (25). N. gonorrhoeae contains several genes known to protect against HP-mediated cellular damage, including the gene encoding high levels of the HP-detoxifying enzyme catalase (26–29, 118, 119). However, many of the genes transcriptionally induced by HP are not protective against HP (24, 30). The apparently large number of genes that respond to HP, coupled with the seemingly minor role of N. gonorrhoeae killing by PMNs through oxidative means during infection, suggests there are additional functions for HP-responsive genes during infection that do not directly relate to protection from oxidative killing. We have proposed that, since HP is a ubiquitous signaling molecule in nature, N. gonorrhoeae might respond to HP signaling during infection (31–33).

As a host-restricted organism, N. gonorrhoeae does not encounter the broad range of environmental conditions that non-host-restricted bacteria do but does have the ability to adapt to many environmental conditions encountered within the human host during the course of infection. After transmission and adherence to the epithelial mucosa, N. gonorrhoeae must adapt to various pH levels found in the male and female genital tracts and to various levels of oxygen content in these different environments and of availability of metals and other nutrients, as well as to the host innate immune response. Adaptation to the host genital mucosa requires N. gonorrhoeae to sense and respond to these variations in oxygen and nutrient concentrations, pH, and the presence of antimicrobial molecules in this environment. In this context, HP may serve as an environmental signaling molecule for N. gonorrhoeae. We hypothesize that, during interactions with macrophages and epithelia or after neutrophil influx, HP signals to N. gonorrhoeae that the host is mounting an inflammatory response. N. gonorrhoeae then instigates an adaptive transcriptional program to adapt to this stage of infection. In support of this hypothesis, it has been shown that N. gonorrhoeae bacteria pretreated with HP have enhanced survival with respect to human PMNs and that the enhanced survival is independent of ROS production (5). To understand the extent to which N. gonorrhoeae genes are regulated by HP, we exposed the bacteria to sublethal amounts of HP and characterized the HP-induced N. gonorrhoeae transcriptome.

To fully characterize the N. gonorrhoeae transcriptional response to HP, we performed RNA sequencing (RNA-Seq) to profile changes to the N. gonorrhoeae transcriptome after exposure to sublethal amounts of HP and differential RNA-Seq (dRNA-Seq) to characterize transcriptional start sites (TSSs). We used this information to annotate HP-responsive operons and showed that the changes in the transcriptome are specific for peroxides and a representative subset of the transcriptome is not responsive to other prominent ROS found in PMNs. We compared the HP transcriptome to those reported under other environmental conditions, such as anaerobiosis or iron availability, and to regulons controlled by specific transcription factors. These analyses show a vigorous and complex response of N. gonorrhoeae to sublethal HP treatment that is mediated by an array of transcription factors and significantly overlaps many of the previously identified transcriptional responses reported for other conditions. These results support the idea that N. gonorrhoeae uses variations in HP levels as a signal for different stages of infection and may indicate new factors involved in the infection process.

## RESULTS AND DISCUSSION

### Outline of experimental design.

**FIG 1:**
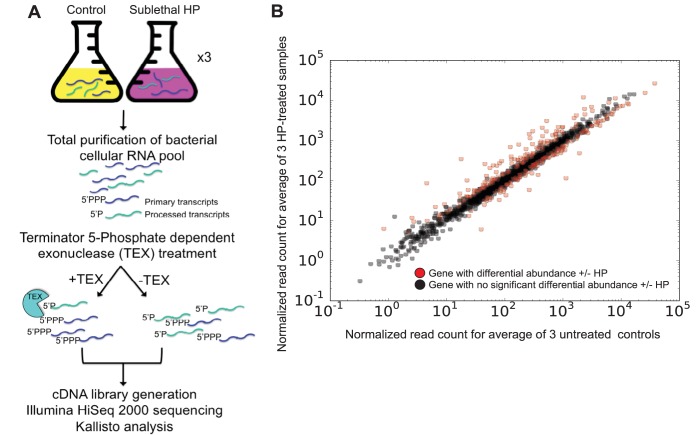
N. gonorrhoeae bacteria treated with sublethal HP show a robust transcriptional response, with 293 genes differentially regulated. (A) RNA-Seq experimental design. N. gonorrhoeae is grown in liquid culture to mid-log phase in three separate experiments and treated with sublethal HP or left untreated. Total RNA is pooled, purified, and prepared for sequencing. For the differential RNA-Seq portion of the experiment, total RNA is split and one portion treated with terminator exonuclease (TEX), enriching for primary transcripts and allowing global annotation of transcriptional start sites (TSSs). (B) Plot of HP treatment versus control transcript levels. A total of 293 genes showed significantly differential transcript abundance between HP-treated and untreated conditions. Each dot on the scatter plot denotes an individual gene in the N. gonorrhoeae genome, with black circles indicating genes without significantly different transcript abundance between conditions, and red circles indicating genes with significant differences in transcript abundance. The *x* axis shows transcript abundance values for the three averaged untreated control samples, while the *y* axis shows transcript abundance values for the three averaged 15 mM HP-treated samples.

In order to investigate a global transcriptional response to HP, we treated exponentially growing N. gonorrhoeae with a sublethal amount of HP. Sublethal concentrations were determined by quantifying the amount of HP that resulted in lethality of less than or equal to 10% killing in CFU levels per milliliter. This HP dosage ensured that we could record the maximal transcriptional response to oxidative damage while ensuring that a majority of the culture was still viable. This level was not meant to replicate a physiological level of HP encountered during infection since the concentration of HP varies considerably from levels that do not kill to levels within a vacuole within an Opa-stimulated PMN that are lethal (34–38). We tried several combinations of growth conditions, levels of HP, and lengths of HP treatment (data not presented) and determined that 15 mM HP treatment for 15 min was consistently sublethal under these growth conditions. Following HP treatment, total HP-treated and untreated control RNA was harvested (Fig. 1A) using a protocol that resulted in abundant, high-quality RNA as measured by 23S/16S ratios and Bioanalyzer. Half of each total RNA sample was used for global mapping of TSSs by dRNA-Seq (39, 40). Sequencing reads were filtered for quality and mapped to the N. gonorrhoeae genome using kallisto, and transcript abundances were quantified using Sleuth (41). kallisto differs from other read mappers in that it rapidly maps reads using pseudoalignment, while preserving accuracy (42). Read depth ranged from 17.0 to 37.8 million reads (see Table S2 in the supplemental material). Analyzing differential transcript abundance by comparing sublethal HP-treated samples to untreated samples, biological replicates showed high consistency between replicates from the same conditions, with an *R*^2^ value of ∼0.99. A total of 293 genes showed differential transcript abundance (false-discovery-rate [FDR; *q*] value, <0.05 [FDR-corrected Wald test]) in HP-treated samples compared to untreated controls (Fig. 1B). A total of 150 genes showed an increase in transcript abundance compared to untreated controls, while 143 genes showed a decrease in transcript abundance compared to untreated controls (Table S3).

These results confirm previous microarray results showing that N. gonorrhoeae mounts a robust response to sublethal HP treatment (24), though there are many differences between these results and the previously reported results. The previous study identified a total of 150 genes that showed differential transcript abundance upon HP exposure, with 75 upregulated and 80 downregulated (24), while this study identified nearly twice the number of HP-responsive genes. A total of 69 genes showing differential transcript abundance overlapped between the studies, while 224 of the genes reported in this study were not found in the previous study. Differences between the previous microarray study and this RNA-Seq study may have resulted from differences in experimental conditions, such as the 5 mM HP concentration used in the previous study versus the 15 mM concentration used in this study, in addition to the relative levels of sensitivity of microarray analysis and next-generation sequencing. RNA-Seq is better able to detect changes in expression in smaller genes and genes that are expressed at a low level (43).

**FIG 2:**
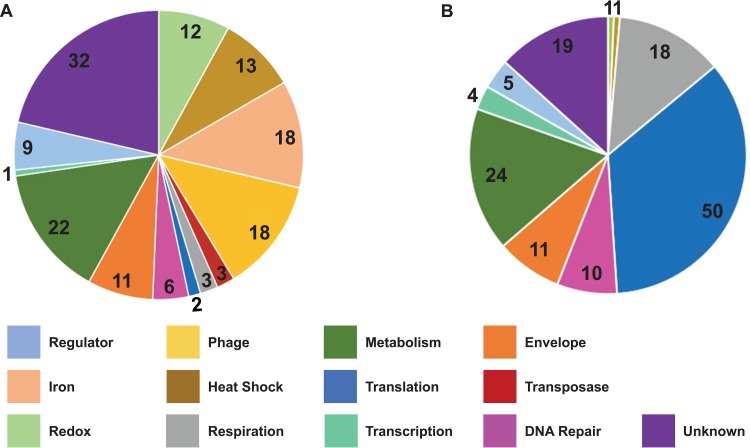
HP-regulated gene products belong to 13 major biological functional categories. A total of 293 genes were found to differ in transcript abundance between mid-log-phase N. gonorrhoeae liquid culture samples treated with 15 mM HP and untreated samples by RNA-Seq (*P*  ≤ 0.05). These genes were classified according to biological function and grouped into 13 major functional categories: Regulator, Unknown, Iron, Oxidative Damage/Redox-Associated, Phage-Associated, Heat Shock/Protein Folding, Aerobic/Anaerobic Respiration, Metabolism/Biosynthesis, Translation, Transcription, Outer Membrane/Protein Transport, Transposase, and DNA Regulation and Repair. Abbreviated names for these functional categories are shown in the figure key with color labels corresponding to the transcript abundance charts in panels A and B. (A) A total of 150 genes had higher transcript abundance in HP-treated samples than in untreated samples. (B) A total of 143 genes had lower transcript abundance in HP-treated samples than in untreated samples.

**FIG 3:**
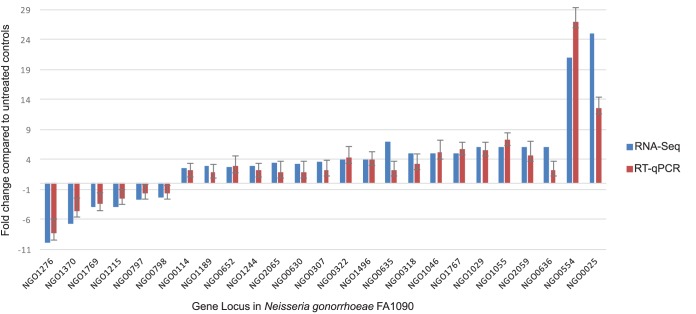
RT-qPCR validation of the RNA-Seq results for a subset of 25 genes. In order to validate the RNA-Seq HP-induced transcriptome, 25 genes were selected from diverse biological functional categories and RT-qPCR was performed on these genes (from new biological replicates) to independently assess gene expression under conditions of sublethal HP exposure. For RT-qPCR validation, liquid N. gonorrhoeae was grown to mid-log phase on three separate days and exposed to 15 mM HP for 15 min or left unexposed. Fold induction values represent the geometric means of results from these three replicates. Black bars denote the RNA-Seq fold induction values (in TPM), while colored bars represent RT-qPCR fold induction values calculated using the 2^ΔΔ^*^CT^* method. Colored bars correspond to biological functional categories.

After classifying the 293 HP-regulated genes into 13 biological functional categories (Fig. 2), we confirmed differential expression of 25 of the 293 genes from a range of functional categories. Validation was confirmed via reverse transcriptase quantitative PCR (RT-qPCR) from RNA isolated from new biological replicates (Fig. 3). These 25 genes represent the 13 functional classes of the entire data set of HP-regulated genes (Fig. 3). Of these 25 genes, 19 had increased transcript abundance in the RNA-Seq experiments, and the RT-qPCR assay confirmed that they had increased transcript abundance after sublethal HP treatment. The six genes that had decreased transcript abundance in RNA-Seq also showed decreased transcript abundance in the RT-qPCR experiments. It should be noted that the magnitudes of the changes often differed between the RNA-Seq and RT-qPCR data, since pairs of RT-qPCR primers detect relative abundance levels on a certain ∼150-bp portion of each gene, whereas RNA-Seq measures transcript abundance through reads averaged across the total length of each gene. Analysis of the 25 selected genes by RT-qPCR showed that the RNA-Seq results reflected reproducible changes, and the RNA-Seq results were also used to test the effect of other oxidants on transcriptional changes.

### Analysis of transcriptional start sites (TSSs).

**FIG 4:**
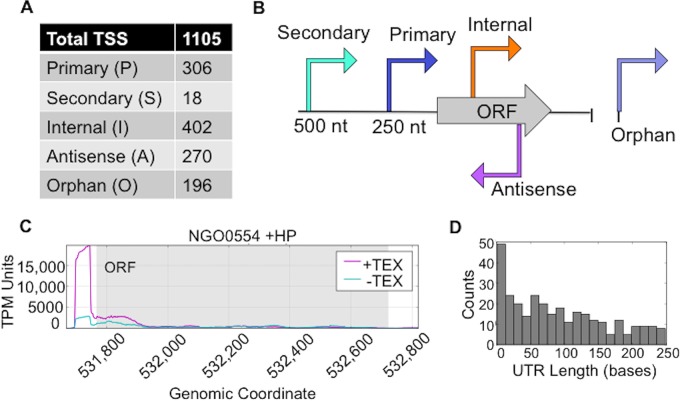
Global annotation of transcriptional start sites (TSSs). The total RNA pool was split equally, and one portion was treated with terminator exonuclease (TEX), which enriches for primary transcripts, creating a characteristic 5′ plateau indicative of a gene’s TSS, as exemplified for NGO0554 in panel C. (A) A total of 1,105 transcriptional start sites were annotated across the N. gonorrhoeae FA1090 genome and assigned to the following 5 categories of TSS: Primary, Secondary, Internal, Antisense, and Orphan. (B) Criteria used to categorize TSSs. Primary (P) TSSs were defined as those within 250 nt or fewer of a gene’s annotated open reading frame (ORF). Secondary (S) TSSs were defined as those within 500 nt or fewer of a gene’s annotated ORF when a primary TSS was present. Internal (I) TSSs were those within an annotated ORF, and antisense (A) TSSs were those within 100 nt or fewer of an annotated ORF on the opposite strand. Orphan TSSs were any that did not meet the aforementioned criteria. (C) Representative example of a characteristic 5′ plateau indicative of the NGO0554 gene’s TSS. The purple line denoting transcript abundance represents TEX treatment (+TEX). The blue line denotes transcript abundance for the TEX-untreated sample (-TEX). The purple line abundance is higher in the 5′ region than the blue line abundance, indicating 5′ enrichment. (D) Global TSS locations relative to annotated ORFs were used to generate this histogram representing the distribution of 5′ untranslated region (UTR) lengths in FA1090.

Differential RNA-Seq (dRNA-Seq) enables global annotation of transcriptional start sites by enriching for primary transcripts when the total cellular RNA pool is treated with terminator exonuclease (TEX) (39). We performed dRNA-Seq in tandem with RNA-Seq (Fig. 1B). Results from the global TSS annotation are presented in Fig. 4A. We established criteria for categorizing the total TSS to compare with other dRNA-Seq studies (Fig. 4B). Primary (P) TSSs were defined as those within 250 nucleotides (nt) or fewer of a gene’s annotated open reading frame (ORF). Secondary (S) TSSs were defined as those within 500 nt or fewer of a gene’s annotated ORF when a primary TSS was present. Internal (I) TSSs were those within an annotated ORF, and antisense (A) TSSs were those within 100 nt or fewer of an annotated ORF on the opposite strand. Orphan TSSs were any that did not meet the aforementioned criteria. TSSs were manually annotated using Integrative Genomics Viewer (IGV) by scanning the RNA-Seq reads for characteristic 5′ enriched plateaus (Fig. 4C). Genes with 5′ plateaus that showed a threshold of abundance enriched 2-fold or more in TEX-treated samples compared to untreated samples were annotated as TSSs. A blind analysis was performed individually by two people. Total TSS locations were used to map the distribution of 5′ untranslated region (UTR) lengths in FA1090 (Fig. 4D). A total of 1,105 TSSs were annotated for FA1090 in this study, similarly to the 1,351 TSSs reported for N. gonorrhoeae strain MS11, which has a larger genome (44).

### HP-regulated operon annotation.

**FIG 5:**
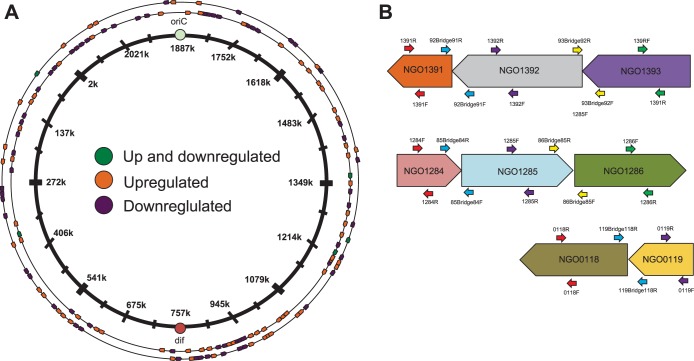
A significant portion of the transcriptional response to HP occurs in operons. (A) HP-induced operon annotation. The 2.1 Mb circular N. gonorrhoeae genome is depicted as a map, with both strands of DNA represented on the outer side of the map and the total of 233 operons represented by the different colored arrows. Operons containing genes that showed increased transcript abundance are colored in orange, while operons containing genes that showed decreased transcript abundance are purple. Five operons showed a mixture of genes that showed increased and decreased abundance, and these are shown in green. (B) Illustration of primer design for select operon validation by RT-qPCR. NGO-1391-1393, a three-gene operon, contains a mixture of genes showing increased transcript abundance, decreased transcript abundance, and no change in transcript abundance (quantified in Table S3). qPCR primers were designed to amplify products internal to each of the three genes, as well as those spanning the sets of genes. If a spanning operon pair gave a *C_T_* value above the value determined for the no-RT control for that pair, it was considered to contain sufficient significant transcript abundance to be called an operon, as detailed in Materials and Methods.

Many bacteria respond to changing environmental conditions through changes in gene expression mediated by operons, which are groups of genes transcribed together from a single promoter. To determine the extent to which the transcriptional response to sublethal HP in N. gonorrhoeae was controlled by operons compared to individual genes, we performed manual annotation of operons within the set of 293 HP-responsive genes. Of the 293 genes showing differential transcript abundance, 233 genes belonged to a total of 140 operons (Fig. 5A). Operon size ranged from 2 to 16 genes, with an average size of 3.5 genes. A total of 87 of the regulated operons contained only one HP-responsive gene, 29 contained a mixture of HP-responsive and HP-nonresponsive genes, and all of the genes in 22 operons showed differential transcript abundance in response to HP. A total of 63 operons were downregulated, and 69 were upregulated. No spatial patterns were observed regarding genomic location, with the HP-responsive operons distributed around the genome, with no obvious clustering. Interestingly, a small number of operons contained genes that were both upregulated and downregulated by HP. This could have been due to differential RNA decay, as has been observed previously in polycistrons in Escherichia coli (45).

To validate our manual operon annotation, three operons were investigated via RT-qPCR (Fig. 5B). The validated operons represent the different types of operons observed by manual analysis. The first, N. gonorrhoeae (NGO)-1391 to NGO-1393, a three-gene operon, contains a mixture of genes showing increased transcript abundance, decreased transcript abundance, and no change in transcript abundance. These validated results show that transcriptomic changes that occur in response to peroxide are not solely mediated by transcriptional regulation. We presently do not know whether differential RNA processing or another form(s) of RNA stability is responsible for the nonuniform transcript abundance in some operons.

### Analysis of the biological functions of the HP-induced transcriptome.

In order to better understand the system-level response of N. gonorrhoeae to HP, we classified all 293 genes showing differential transcript abundance into 1 of 13 major biological functional categories based on information gathered from existing N. gonorrhoeae literature, the UniProt database, and NCBI BLAST homology (41, 46, 47) (Table S3). We found that 52 of 293 genes (17.7%), including 32 genes with increased transcript abundance and 19 genes with decreased transcript abundance, were of unknown biological function (Fig. 2). For the remaining genes, the following sections discuss subsets of our data set that overlap known regulatory networks, transcriptional regulators that were affected by HP exposure, and several of the primary systems that we found to be most significantly involved in the transcriptional response to HP. However, as discussed next, some of the HP response was clearly due to transcriptional regulation.

### HP-induced transcriptional regulators and interaction with major N. gonorrhoeae regulons.

The 293-gene HP-responsive data set comprises 14% of the 2,157-gene FA1090 genome. As a host-restricted pathogen, N. gonorrhoeae has many relatively small and overlapping known regulatory pathways that respond to differing concentrations of oxygen and iron and differing levels of antimicrobial peptides and pH within its urogenital cellular niche. A meta-analysis was performed to determine which of the 293 HP-responsive genes described in this study overlapped the regulatory networks described in 20 other studies (24, 48–66) (Table S3). While the HP-regulated transcriptome did not show total overlap with any other major regulatory networks, a subset of genes regulated by HP did overlap subsets of genes that were regulated by iron or oxygen levels or by characterized transcriptional regulators (Tables S4 and S5).

A total of 14 transcriptional regulators showed differential transcript abundance when exposed to sublethal HP, with 9 regulators showing increased abundance and 5 regulators showing decreased abundance. Interestingly, 117 genes found in this study had not previously been reported to be part of any known major regulatory network, underscoring the depth of the pool of unknown HP-responsive transcriptional regulation data yet to be uncovered.

### HP-transcriptome overlap of the iron and Fur regulons and HP-regulated genes involved in iron transport and homeostasis.

Iron homeostasis is crucial to N. gonorrhoeae survival and pathogenesis. Pathogenic *Neisseria* bacteria require iron for survival inside the human host and scavenge iron from human transferrin, lactoferrin, and heme directly, while the host actively sequesters iron as a form of nutritional immunity (67, 68). Other studies have observed overlap of the N. gonorrhoeae HP transcriptome and the sizable network of genes affected by iron and oxygen levels (24, 57). In this study, we detected that sublethal HP exposure induced the gene encoding Fur, a global transcriptional regulator that regulates iron homeostasis in response to intracellular iron levels. Sublethal HP also induced IscR, the regulator that controls the *isc* Fe-S cluster biosynthesis operon. These results suggest that HP influences the two major regulators involved in iron homeostasis. We compared the HP-regulated transcriptome data to data from other transcriptional profiling studies investigating the transcriptional response of N. gonorrhoeae to differing iron levels and the presence of the Fur iron-responsive regulator (49, 51, 60) (Tables S4 and S5). A set of 48 HP-regulated genes were also affected by iron levels, with 25 of these genes thought to be part of the Fur regulon (Table S4). A strong correlation with the direction of regulation (upregulation or downregulation) was observed for this iron- and Fur-overlapping gene set, as the majority of HP-induced genes that were iron repressed were upregulated in the presence of HP. Similarly, the majority of HP-repressed genes were iron induced. It is possible that these HP-regulated genes are under the direct regulation of Fur, which may become oxidized and inactivated by HP (69). It is also possible that these HP-regulated genes are indirectly regulated by Fur, as Fur is known to regulate other transcriptional regulators (60).

A total of 18 genes previously shown to be associated with iron homeostasis showed an increase in transcript abundance compared to untreated controls. Of these, eight are thought to be regulated by Fur (49). In addition to damaging Fe-S cluster-containing enzyme centers, HP reacts with free iron inside bacteria to produce highly reactive hydroxyl radicals that directly damage proteins and deplete intracellular free iron pools (70). As such, iron-associated genes that respond to HP belong to the following two main categories: (i) those that act to tightly regulate iron transport, maintaining the intracellular free iron pool, and (ii) those involved in biosynthesis of Fe-S cluster-containing enzyme centers. Iron is transported across the outer and cytoplasmic membranes by Ton-B-dependent transporters, and genes like *tbpA*, *tbpB*, *hpuB*, *fetA*, *fetB*, *tonB*, *exbB*, *exbD*, *tdfG*, *and efeO*, all involved or putatively involved in iron transport (71–76), showed increased transcript abundance.

In addition to affecting iron transport and acquisition, increased intracellular HP levels have been shown to affect proteins containing Fe-S clusters. These chemically versatile structural protein centers contain ensembles of iron and sulfide, are found ubiquitously throughout the bacterial cell, are responsible for a variety of enzymatic activities, and are chemically vulnerable to oxidation (77). N. gonorrhoeae orthologs of the well-studied E. coli system of Fe-S cluster assembly and repair, *iscS*, *iscA*, *iscU*, *and erpA*, showed increased transcript abundance, as did the biosynthesis master regulator gene *iscR* (77). These data illustrate a complex interplay between the HP-sensing and iron-sensing regulons of N. gonorrhoeae and suggest the hypothesis that iron availability and protection from oxidative killing may have other functional interactions.

### HP-transcriptome interaction with gene networks affected by oxygen concentration and HP-regulated genes involved in cellular respiration.

Previous reports have also identified genes whose expression is affected by both the presence of HP and oxygen availability (57). Sixty-five genes that had differential transcript abundance when N. gonorrhoeae bacteria were grown anaerobically overlapped our HP-regulated data set. Similarly to our comparisons performed with iron-regulated transcripts, the subset of HP-regulated genes that were also anaerobic regulated showed a strong correlation with respect to the direction of regulation. The majority of HP-induced genes that overlapped were induced under anaerobic conditions and the vast majority of HP-repressed genes were repressed under anaerobic conditions (Table S4).

Electron transport chains are necessary in bacteria to drive metabolism, maintain proton motive force, and provide electron transport for ATP synthesis (78). During the course of infection, N. gonorrhoeae is subjected to oxygen limitation inside the host urogenital tract (4, 57) and evidence shows that anaerobiosis is a physiologically significant state for N. gonorrhoeae during human infection (54, 57, 64). Three transcriptional regulators involved in oxygen sensing, as well as 20 genes involved in cellular respiration, denitrification, and anaerobiosis, displayed differential transcript abundance in response to HP exposure. The gene *aniA*, which controls the N. gonorrhoeae denitrification system required for anaerobic growth, as well as other genes involved in denitrification showed decreased transcript abundance. The genes encoding the nitrate-responsive two-component regulatory system NarQ-NarP showed decreased transcript abundance. N. gonorrhoeae NarQ-NarP activates the *aniA* nitrite reduction gene but does not do so in response to any known stimulus. Studies profiling a *narP* mutant have found a small regulon focused on denitrification (64, 79). The gene encoding oxygen-sensing transcriptional regulator FNR showed increased transcript abundance. Whereas FNR affects transcription of more than 100 operons in E. coli, only 20 genes have been shown to be regulated by FNR in N. gonorrhoeae (54), including the *aniA* and *norB* anaerobic operon and *ccpR* cytochrome *c* peroxidase genes. *ccpR*, a cytochrome *c* peroxidase, has been previously shown to protect N. gonorrhoeae from HP but not from reactive nitrogen species (RNS) (118). A subset of six genes regulated by FNR overlapped genes regulated by HP, with correlation with respect to the direction of regulation. HP-induced genes have also been shown to be activated by FNR, while HP-repressed genes have been shown to be repressed by FNR. These data suggest that HP affects oxygen-sensing regulators and influences the regulation of cellular respiration at many levels. It is possible that FNR may be inactivated by oxidation under conditions of HP exposure.

It has been shown that anaerobically grown Saccharomyces cerevisiae bacteria are hypersensitive to low doses of HP (80), implying the possibility that downregulation of anaerobic respiration genes can serve to protect N. gonorrhoeae from oxidative damage. In addition, oral dental niche bacteria that use HP as an antimicrobial to compete with other bacteria for niche colonization downregulate HP synthesis in response to anaerobic conditions, implying that these two pathways may be commonly regulated opposite one another (81). It has been observed that when grown microaerobically, the nitrogen-fixing environmental Gram-negative bacterium Rhodobacter sphaeroides switches to high-oxygen metabolism after exposure to HP (82). It is similarly possible that downregulation of anaerobic respiration serves to protect N. gonorrhoeae from HP exposure through protection against oxidative damage. Whether HP may adaptively signal N. gonorrhoeae to undergo a transcriptional program suitable for the different types of respiration required for different various stages of infection and, if so, what role this might play during infection remain to be determined. Interestingly, it has been suggested that the N. gonorrhoeae denitrification pathway’s reduction of environmental NO, required for anaerobic respiration, may have immunomodulatory effects on the host through immune cell signaling via NO concentration, specifically affecting the incidence of asymptomatic disease (79).

### HP-transcriptome interaction with the RpoH regulon and HP-regulated genes involved in protein folding and homeostasis.

Radicals produced by HP can directly interact with proteins, causing damage and misfolding, and the altered intracellular redox conditions caused by increased HP concentration can cause bonds within proteins to be oxidized, also contributing to protein misfolding (83). In addition, the transcriptional regulatory circuits that respond to heat shock and general cellular distress in N. gonorrhoeae, such as those involving genes controlled by protein homeostasis regulator RpoH, have been shown to overlap the transcriptional response to HP (53). Indeed, 17 genes belonging to the known RpoH regulon showed differential transcript abundance when exposed to sublethal HP in this study (Tables S4 and S5). All 15 overlapping HP-induced genes were RpoH or heat shock activated, and two HP-repressed genes were RpoH or heat shock repressed. A total of 12 genes associated with heat shock and protein homeostasis showed an increase in transcript abundance under conditions of sublethal HP levels. Nine of those genes were previously shown to be part of the RpoH regulon (*dnaJ*, *dnaK*, *grpE*, *groES*, *clpB*, *lon*, and *secB*, which encode chaperones that stabilize protein conformation and refold misfolded proteins [84, 85], *hscB*, which [together with *hscA*] encodes cochaperones to Hsp70 [86, 87], and *htpX*). Due to HP’s ability to produce free radicals and cause protein damage and misfolding, it may activate RpoH-mediated pathways to maintain protein homeostasis.

### HP-transcriptome interaction with the MtrR and MpeR regulons.

The gene encoding transcriptional regulator MpeR showed increased transcript abundance in response to sublethal HP treatment. MpeR has been shown to directly repress *mtrR*, encoding the MtrR repressor of the MtrCDE efflux pump, thus increasing efflux in response to low iron levels in the cell (58). These two regulators have no previous known function contributing to regulation under oxidative conditions. *mtrR* did not show decreased transcript abundance in this study, possibly owing to our experiment being performed on N. gonorrhoeae in mid-log phase under iron-replete conditions, while MpeR repression of *mtrR* occurs in stationary phase under iron-depleted conditions. Comparing the sublethal HP-regulated transcriptome to the known MtrR and MpeR regulons, two subsets of overlapping genes were observed, with a strong correlation observed for the direction of regulation for both regulators. A total of 14 HP-regulated genes regulated by HP are regulated by MpeR, while 12 HP-regulated genes are regulated by MtrR. Twelve genes were induced by HP and repressed by MpeR, while eight genes were induced by HP and repressed by MtrR. As discussed above, four heat shock-related and protein-folding-related genes of the MpeR regulon (*clpB*, *grpE*, *prlC*, and *groES*) were increased in transcript abundance. An increase in the transcript abundance in these protein-folding-related and homeostasis-related genes may indicate a mechanism through which MpeR responds to HP during infection. MtrR is known to directly repress *rpoH*, so MpeR repression of MtrR could affect gene expression in an RpoH-mediated manner, possibly with *mtrR* repression occurring below the level of detection under these conditions. Alternatively, it may indicate a previously unknown overlap of transcriptional regulation through RpoH and regulation through MpeR. Since efflux is a critical process for antibiotic resistance, it is possible that the HP levels influence the sensitivity of N. gonorrhoeae to antimicrobials.

### Effect of sublethal HP on other transcriptional regulators.

In addition to those already discussed, three transcriptional regulators showed decreased transcript abundance in response to sublethal HP. Two noncanonical regulators, *ngoAX* and *dksA*, two N. gonorrhoeae genes that regulate transcription without directly binding to DNA, showed decreased transcript abundance. *ngoAX* encodes a methyltransferase (NgoAX) shown to globally regulate gene expression, biofilm formation, interaction with epithelial cells, and growth, resulting in decreased transcript abundance (52). Five of the 121 genes known to be regulated by NgoAX also showed differential transcript abundance after HP treatment (52). The second noncanonical regulator gene was a homolog of *dksA*, a gene encoding an RNA polymerase (RNAP) binding protein that regulates the RNAP reaction to ppGpp and rRNA transcription (88). It is not known if ppGpp levels are linked to coordination of an adaptation program to oxidative conditions, but guanine nucleotides are the most common target of oxidation (89).

In addition to those already described, seven transcriptional regulators showed differential transcript abundances in response to HP. Among the total of 14 regulators, *fur*, *iscR*, *mpeR*, *lexA*, and *lrp* were detected by a previous microarray study that exposed N. gonorrhoeae to HP (24). In addition to those regulators already discussed, the following four regulators showed increased transcript abundance in response to HP: *lexA*, *marR*, *npr*, and *gntR*. *lexA* encodes a homolog to E. coli SOS response regulator LexA. LexA has been shown to regulate three N. gonorrhoeae genes, namely, itself, *recN*, and a gene of unknown function, NGO1428, all of which showed increased transcript abundance in this study (90). This result suggests that HP regulation of these genes was dependent only on LexA, since the DNA-binding ability of LexA is inhibited by the oxidation of a cysteine residue by HP (90). Another gene, *marR*, encoding a protein belonging to the multiple antibiotic resistance regulator (MarR) family of transcriptional regulators, showed increased transcript abundance with no previously known adaptation to oxidative conditions in N. gonorrhoeae. MarR regulators are repressors whose DNA-binding activity is attenuated by oxidation of certain cysteine residues (91), indicating a possible mechanism for the HP-induced *marR* transcript level.

### A subset of cryptic prophage genes is induced by HP.

Putative phage repressor *npr* showed increased transcript abundance in response to HP. Little is known about the function of any of these phage-related genes in N. gonorrhoeae. *npr*, a XRE phage repressor, resides in a genomic region thought to be an incomplete prophage island incapable of forming functional particles and has been previously shown to regulate genes within the NGO0460-NGO0463 phage locus (92). A mutant in this repressor showed increased adhesion to epithelial cells in tissue culture and increased colonization of a mouse mucosal model (92), suggesting that this transcriptional regulator has also been coopted to regulate bacterial genes, though the identity of the genes in this putative regulon remains unknown.

The most unexpected result from this study was the increased transcript abundance upon HP exposure of 19 phage-associated genes, none of which were reported in the previous microarray study (24). Induction of lytic phage by hydrogen peroxide has been observed in other Gram-negative bacteria (49, 63, 93, 94). A study performing RNA-Seq on clinical gonococcal exudates from women found increased transcript abundance in seven phage-associated genes in cervical exudate (95). Two of these genes, a repressor *npr* gene and NGO0509, a gene of unknown function, were regulated by HP in our study or had orthologs that were regulated by HP in our study (Table S6). A new study comparing *in vivo* gonococcal gene expression levels between men and women found that phage-associated genes were expressed in bacteria isolated from both sexes. Two of the genes found in this study, repressor gene *npr* and gene of unknown function NGO1030, are regulated by HP or have orthologs shown to be regulated by HP (96). A study in E. coli showed that the presence of cryptic prophage genes in the genome protected bacteria against challenge by HP, antibiotics, and osmotic and acid stress (97). Taking the data together, the HP induction of some phage genes supports the idea of a potential role for these in N. gonorrhoeae infection.

### Genes involved in translation showed decreased transcript abundance.

A total of 51 genes involved in maintenance of translation showed a decrease in transcript abundance under conditions of sublethal HP exposure, representing a much larger effect on genes involved in translation than was seen in the previous microarray study. A total of 32 of these genes code for ribosomal structural proteins involved in subunit assembly. In addition, five genes involved in tRNA decoding of codons during translation showed decreased transcript abundance, as did four genes involved in charging tRNA with amino acids. HP exposure of E. coli results in reduction of protein synthesis, specifically through enzymatic degradation of tRNAS (98). Oxidative stress also induces mistranslation in E. coli, which can be protective against heat shock, acidic conditions, high osmolarity, and antibiotic exposure (99, 100). These results show that HP has an inhibitory effect on translation that most likely indicates a reduction in growth. Whether this effect on translation is a cause of or an effect of growth reduction remains to be determined.

### Genes involved in oxidative damage protection and redox homeostasis showed increased transcript abundance.

In exposing N. gonorrhoeae to sublethal HP, increased transcript abundance for genes known to protect against oxidative damage and maintain redox homeostasis was expected. Twelve genes that contribute to these functions were detected by our study (Table S3). Among the genes with the highest fold increase in transcript abundance were *katA* and *msrAB*, genes that have been shown to protect N. gonorrhoeae against HP and other ROS present in the PMN phagosome (28, 101). Several genes known to be involved in maintaining redox homeostasis under oxidative conditions were detected, including *gor*, a glutathione (GSH) oxidoreductase gene that maintains a reduced cellular pool of chemical scavenger GSH (27, 66), and many genes encoding members of the glutaredoxin, thioredoxin, and peroxiredoxin protein families (*prx*, *osmC*, *grx4*, *grx3*, and *trx*). *katA*, *prx*, and *gor* are members of the OxyR regulon; however, the gene encoding OxyR was not affected by HP exposure in this study or in the 2005 microarray study. This result may indicate that HP activation of OxyR occurs at the posttranslational level through reversible disulfide bond formation, as is the case for E. coli (102).

### Genes involved in DNA replication and repair were affected by HP.

Expectedly, as DNA is a target for ROS in bacteria, 16 genes involved in DNA replication and repair showed differential transcript abundance levels. Of these, 6 genes showed an increase in abundance and 10 showed a decrease. Free radicals directly attack DNA purine and pyrimidine bases, as well as sugar moieties, creating single-strand and double-strand breaks in the DNA backbone. Other products of free radical damage to DNA include base and sugar adducts and DNA cross-links to other molecules, creating lesions that can stall replication (103).

### Many genes involved in N. gonorrhoeae physiology were affected by HP.

In addition, 46 genes involved in general cellular metabolism or biosynthesis were affected, with 22 genes showing increased abundance and 24 genes showing decreased abundance. The main categories affected by HP exposure in metabolism were amino acid synthesis, pyruvate metabolism, tricarboxylic acid (TCA) cycle reactions, and glycolysis. Interestingly, *relA*, the gene encoding a ribosome-associated (p)ppGpp synthetase (PSI), showed increased transcript abundance. (p)ppGpp is an alarmone, a compound that accumulates in the cytoplasm of cells starved for amino acids, controlling a pleiotropic response known as the stringent response. During the stringent response, macromolecular synthesis, metabolism, and rRNA and tRNA synthesis are downregulated and *rpoH* is upregulated. In N. gonorrhoeae, RelA alone is responsible for the accumulation of (p)ppGpp in cells starved for serine (104). An Enterococcus faecalis *relA* mutant grew better in 1 mM HP but was more sensitive to 45 mM HP than the parental strain (105). As HP can affect protein homeostasis and cellular transport, it was expected that envelope-associated proteins would show differential transcript abundance levels in response to HP. A total of 21 envelope-associated genes showed differential transcript abundances. Eleven of these genes encoded adhesins or outer membrane proteins, the majority of which showed a decrease in transcript abundance.

### The HP-induced transcriptome overlaps the organic peroxide transcriptome but not with bleach or superoxide.

**FIG 6:**
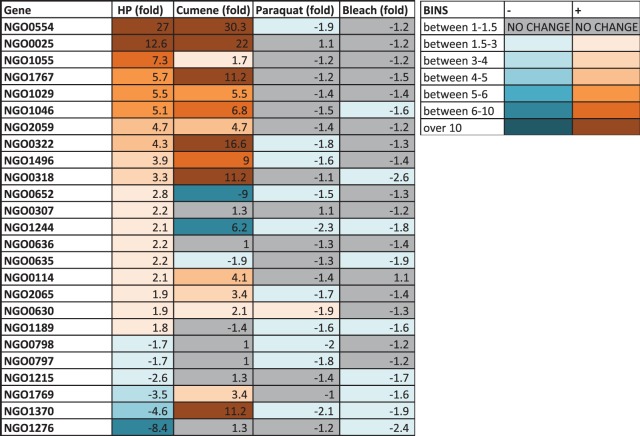
Transcriptional responses of 25 representative genes to other oxidants. Results are shown for 25 genes selected to investigate the specificity of the HP-induced transcriptional response via RT-qPCR. Liquid N. gonorrhoeae was grown to mid-log phase and treated with sublethal amounts of paraquat, HOCl, or cumene hydroperoxide for 15 min, after which total RNA was purified and RT-qPCR was performed. Numerical values represent the geometric mean fold change compared to the levels seen with the *omp3* housekeeping gene for three biological replicates for HP and cumene hydroperoxide and two replicates for paraquat and HOCl. The key to the right of the figure denotes the gradient colors corresponding to bins representing the magnitude of fold change values. Genes that showed no differential expression between treated and untreated samples are shown in gray, downregulated genes are shown in blue, and upregulated genes are shown in orange.

During infection, N. gonorrhoeae encounters different types of reactive oxygen species (ROS) in addition to HP. N. gonorrhoeae bacteria produce various ROS as by-products of their own metabolic and redox homeostasis and also encounter ROS produced by resident commensal lactobacilli in the surrounding genital tract. In addition, N. gonorrhoeae encounters host-derived ROS produced by epithelial cells, macrophages, and PMNs. In order to determine the specificity of the HP-induced transcriptome and whether HP-responsive genes also respond to other prominent ROS encountered during infection, we exposed N. gonorrhoeae in liquid medium to sublethal amounts of paraquat, HOCl (a proxy for O_2_^-^), and an organic conjugated peroxide, cumene hydroperoxide. Parameters similar to those set for HP were used to define sublethal levels of these oxidants. RT-qPCR was performed for a representative set of 25 HP-responsive genes after exposure to cumene hydroperoxide, paraquat, or HOCl (Fig. 6). Treatment with sublethal levels of cumene hydroperoxide produced similar transcriptional responses with 14 of 25 genes. This result shows that some of the response is shared between inorganic and organic peroxides. The lack of response for the 11 other genes is likely the result of differences in localization, intracellular concentration, or regulatory networks responding to the organic peroxide. Paraquat and HOCl produced no transcriptional response for any of the 25 genes tested. A full RNA-Seq study of these two oxidants will be required to determine whether there is a subset of HP-regulated genes that are responsive to these agents. In order to test whether pretreatment with sublethal HP resulted in increased survival of environmental challenges encountered during infection, we tested whether N. gonorrhoeae treated with 15 mM HP could better survive subsequent lethal challenge with HP, HOCl, paraquat, and nonoxidative antimicrobial peptide LL-37. No increased survival was observed with HP pretreatment (data not shown). We presume either that the complex transcriptional response to HP includes genes that both sensitize and resist these agents or that the protective gene products are already expressed at levels that are high enough to provide maximum protection under these growth conditions.

### Summary.

The results of this study show that there is an unusually robust N. gonorrhoeae transcriptional response to sublethal HP, with a total of 293 HP-responsive genes identified. While all of the responses were consistent in biological replicates and were statistically significant, some of the responses were minor and may not reflect important changes in N. gonorrhoeae physiology. This response was much larger than was reported in the 2005 microarray study, which is a consequence of the fact that the microarray used was less capable of detecting small changes in expression or changes in poorly expressed genes. While expression of several transcriptional regulators was altered, there are 117 differentially expressed genes belonging to no known regulatory pathway, suggesting that there are regulatory pathways of N. gonorrhoeae that are not well characterized. The identification of induced phage-associated genes and the apparent downregulation of the denitrification pathway anaerobiosis-associated genes and translation indicate that there are new N. gonorrhoeae bacterial processes that may contribute to pathogenesis. Moreover, these transcriptional responses are consistent with the hypothesis that host-derived HP can serve as a signaling molecule during infection in addition to its role as an antimicrobial agent.

## MATERIALS and METHODS

### Strains and growth conditions.

N. gonorrhoeae strain GCM77 (106) was used for all experiments. GCM77 is a derivative of strain FA1090 containing a mutation in the *pilE* G4 region preventing pilin antigenic variation (107) and a mutation in the *pilC1* coding sequence preventing pilus-phase variation (108). These two mutations stabilize the piliated state and prevent extensive pilus-phase variation from affecting the results. N. gonorrhoeae was treated with sublethal HP in liquid culture, as opposed to plate growth, to ensure even levels of growth and equal levels of HP treatments within the entire bacterial population. Exponential growth in liquid media was achieved using a three-day protocol as follows. On day 1, N. gonorrhoeae was streaked from freezer stock onto N. gonorrhoeae medium base (Difco) agar plus Kellogg supplement I (22.2 mM glucose, 0.68 mM glutamine, 0.45 mM cocarboxylase) and Kellogg supplement II [1.23 mM Fe(NO_3_)_3_] (109) (GCB+) and incubated at 37°C and 5% CO_2_ for 16 h. On day 2, 20 colonies were picked, used to streak heavy lawns of bacteria on GCB+ on plates that had been freshly poured the day before, and incubated at 37°C and 5% CO_2_ for 10 h. The lawns were collected using sterile Dacron swabs and inoculated into 6 ml of liquid GCB+ with 0.042% sodium bicarbonate (GCBL) to an optical density at 600 nm (OD_600_) of ∼0.05 in 15-ml conical tubes (Sarstedt). These cultures were incubated at 30°C with shaking for 16 h. On day 3, piliated bacteria from these tubes were allowed to pellet at the bottom of the tube. The pellets were collected by the use of a sterile pipette and added into 20 ml of fresh GCBL in a 50-ml conical tube (Sarstedt) and grown for 3 h at 37°C with shaking at 220 rpm. At 3 h, 2.5 ml of culture was added to 22.5 ml of fresh liquid GCBL and grown for another 3 h at 37°C with shaking at 220 rpm. A 50-ml volume of culture was added to 380 ml of fresh GCBL and grown for 3 h at 37°C with shaking at 220 rpm to reach the mid-log phase for HP treatment.

### Determination of sublethal concentrations of ROS reagents.

The sublethal concentration of each ROS reagent tested (HP, paraquat, HOCl, cumene hydroperoxide) was defined by determining the concentration of each reagent resulting in some killing but less than 10% killing after 15 min of treatment at mid-log phase in liquid culture by quantifying CFU per milliliter of liquid culture. Killing curve experiments were repeated a minimum of three times on three different days. The sublethal concentrations used were 15 mM HP, 1 μM paraquat, 1 μM HOCl, and 0.01% (vol/vol) cumene hydroperoxide. Untreated control cultures had the appropriate amount of double-distilled H_2_O added, except for the cumene hydroperoxide-untreated controls, which had 30 μl of 95% ethanol added.

### RNA isolation and differential RNA-Seq preparation.

All water used to make reagents used in RNA isolation was subjected to diethyl pyrocarbonate (DEPC) treatment. After treatment, 500 ml of N. gonorrhoeae in mid-log liquid culture was added to a half-volume of ice in plastic bottles to stop growth and RNase production quickly (110) and was centrifuged at 10,000 rpm for 20 min at 4°C. Pellets were resuspended in 500 μg of ice-cold phosphate-buffered saline (PBS), pelleted, and washed once with 1× ice-cold PBS and then resuspended in 500 μg PBS and stored overnight at −80°C. Lysis was achieved by the addition of 50 μg of 1 mg/ml lysozyme (Sigma), and then samples were flash frozen in liquid nitrogen for 5 min and thawed for 5 min in a 37°C water bath. Samples were treated with 15 μg of 15 mM sodium deoxycholate, and the freeze-thaw process was repeated (111, 112). Insoluble materials were pelleted for 10 min at 15,000 rpm at 4°C in a tabletop centrifuge, and supernatants were treated using a Qiagen RNeasy minikit (catalog no. 74104) according to the manufacturer’s instructions, eluting the total RNA into 50 μg RNase-free H_2_O. RNA was precipitated overnight at −80°C with isopropanol and sodium acetate precipitation. RNA quality was determined by calculating the 23S/16S ratio using a Bio-Rad Experion StdSens kit (catalog no. 7007104).

Half of each HP-treated and each untreated biological replicate RNA sample was enriched for primary transcripts by selective degradation of 20 μl of total RNA with 1 U/μl RNA 5′ monophosphate ends (5-P) by treatment with 5-P-dependent terminator exonuclease (TEX; Epicentre catalog no. TER 51020) for 60 min at 30°C (39). The reactions were stopped with 1 μg of 0.5 mM EDTA (pH 8) and were purified by the use of an RNeasy kit according to the manufacturer’s protocol. Samples were precipitated overnight at −80°C with isopropanol and sodium acetate precipitation. RNA quality was determined by the use of a Bio-Rad Experion StdSens kit.

### RNA sequencing.

Equal amounts of HP-treated RNA, untreated RNA, and TEX-treated RNA were used for library preparation and generation. Quality control, library preparation, rRNA depletion by complementary oligonucleotide depletion, and Illumina sequencing were performed at the University of Chicago Next-Generation Sequencing core facility under the direction of Pieter Faber. rRNA depletion experiments were performed using Ribo-Zero (Epicentre) kits for Gram-negative bacteria. cDNA library preparation was performed using TruSeq kits (Illumina). Single-end 50-bp sequencing was performed using an Illumina HiSeq 2000 system.

### Bioinformatic analysis of sequencing data.

The 3′ adapters were trimmed from raw data reads using fastx_clipper, part of the FastX toolkit (http://hannonlab.cshl.edu/fastx_toolkit) (113). Low-quality reads were filtered using fastq_quality_filter, filtering out reads where more than 50% of bases in the read had a phred quality score of less than 20. We used Bowtie 1.1.0 to rapidly remove sequences aligning to rRNA genes (114, 115). For the remaining reads, we used kallisto (version 0.42.3) for subsequent read mapping to the N. gonorrhoeae genome (NC_002946.2) (42). We built two separate indices from this genome: (i) the subset of annotated coding sequences for differential expression analysis and (ii) the complete genome for visualization and dRNA-Seq analysis. Relevant parameters included flags to indicate single-end sequencing, 100 bootstrap replicates, average fragment length size of 50, and a standard deviation of fragment length size of 10. The “pseudobam” flag was included to create .SAM output files for data visualization, which were subsequently converted to .WIG files for later visualization. Differential expression analysis using the kallisto output was performed using Sleuth (version 0.28.0) (41). Sleuth builds a general linear model to compare expression levels between conditions (in our case, control versus HP-exposed growth conditions) while controlling for technical noise. Reported *P* values are from the sleuth_wt function; all reported differentially expressed genes had a false-discovery-rate (FDR) adjusted *P* value (*q* value) of <0.05, regardless of their fold difference values.

### RT-qPCR analysis of transcript abundance.

For validation of RNA-Seq data and for testing a subset of the transcriptional responses to other ROS reagents, total RNA from N. gonorrhoeae treated with a sublethal concentration of HP, paraquat, HOCl, or cumene hydroperoxide was isolated as described above. Experiments were performed in triplicate on three different days for HP and cumene hydroperoxide and in duplicate on two different days for paraquat and HOCl. RNA (5 μg) was treated with 5 U Promega DNase (M6101) for 30 min at 37°C and purified by the use of an RNeasy kit. cDNA was generated using Invitrogen SuperScript reverse transcriptase (RT) III (18080093), including a no-RT control for each sample. cDNA was purified using a QIAquick PCR purification kit (catalog no. 28104). qPCR primers were designed using Primer BLAST and listed in Table S1 in the supplemental material. RT-qPCR was performed on cDNA using Bio-Rad iQ SYBR green Supermix (catalog no. 170-8880) on a Bio-Rad iQ5 Real-Time Detection system, and the N. gonorrhoeae *omp3* gene was used as a housekeeping gene control (116). Fold change values were calculated using the cycle threshold (2^−ΔΔ^*^CT^*^)^ method, and the values presented represent the geometric means of data from all biological replicate experiments. RT-qPCR assays were performed under conditions of minimum information for publication of quantitative real-time PCR experiments (MIQE) best practices.

10.1128/mSystems.00156-18.1 TABLE S1 Primer sequences used in qPCR validation, operon validation, and ROS specificity experiments. Download Table S1, XLSX file, 0.04 MB. Copyright © 2018 Quillin et al. 2018 Quillin et al. This content is distributed under the terms of the Creative Commons Attribution 4.0 International license.

10.1128/mSystems.00156-18.2 TABLE S2 Raw read statistics determined for three HP-untreated and HP-treated replicates, which were then split and treated with TEX enzyme for a total of 12 samples. These reads were used as the input for kallisto analysis. Download Table S2, XLSX file, 0.04 MB. Copyright © 2018 Quillin et al. 2018 Quillin et al. This content is distributed under the terms of the Creative Commons Attribution 4.0 International license.

10.1128/mSystems.00156-18.3 TABLE S3 Data for 293 HP-responsive genes regarding locus number, gene name, direction of transcriptional response to N. gonorrhoeae, and biological functional categorization and notes on biological functional categorization. Download Table S3, XLSX file, 0.1 MB. Copyright © 2018 Quillin et al. 2018 Quillin et al. This content is distributed under the terms of the Creative Commons Attribution 4.0 International license.

10.1128/mSystems.00156-18.4 TABLE S4 Data regarding overlap of 293 HP-responsive genes from this study and other known regulons from other published profiling studies. Download Table S4, XLSX file, 0.1 MB. Copyright © 2018 Quillin et al. 2018 Quillin et al. This content is distributed under the terms of the Creative Commons Attribution 4.0 International license.

10.1128/mSystems.00156-18.5 TABLE S5 Direction of transcription expression information for main regulons overlapping the sublethal HP-induced transcriptional response from this study. Download Table S5, XLSX file, 0.04 MB. Copyright © 2018 Quillin et al. 2018 Quillin et al. This content is distributed under the terms of the Creative Commons Attribution 4.0 International license.

10.1128/mSystems.00156-18.6 TABLE S6 Analysis of phage-related genes regarding percent homology between paralogous phage genes. Download Table S6, XLSX file, 0.05 MB. Copyright © 2018 Quillin et al. 2018 Quillin et al. This content is distributed under the terms of the Creative Commons Attribution 4.0 International license.

### Genome-wide annotation of transcriptional start sites (TSSs).

N. gonorrhoeae TSSs were manually annotated by scanning of the RNA sequence reads overlaid onto the FA1090 genome sequence using Integrative Genomics Viewer (IGV 2.3.72) for the characteristic 5′ plateaus of TSSs (39, 117) in a blind manner by two different researchers. TSSs were identified by the presence of enriched 5′ plateaus whose increased transcript abundance was greater than 2-fold relative to the TEX-untreated control according to agreement from 2 independent annotators. TSSs were designated primary TSSs (if they were within 250 nt of an annotated open reading frame [ORF] on the same strand) or secondary TSSs (if they were 500 nt upstream of an ORF when a primary TSS was present). TSSs were designated internal TSSs if they occurred within an ORF on the same strand and antisense TSSs if they were within 100 nucleotides (nt) upstream or downstream of an ORF on the opposite strand. TSSs were designated orphan TSSs if they did not meet any of the other criteria (Table S7). A total of 30 TSSs were different under conditions of HP exposure alone.

10.1128/mSystems.00156-18.7 TABLE S7 dRNA-Seq global transcriptional start site annotation. Download Table S7, XLSX file, 0.1 MB. Copyright © 2018 Quillin et al. 2018 Quillin et al. This content is distributed under the terms of the Creative Commons Attribution 4.0 International license.

### Annotation of differentially expressed operons and validation of annotation.

The 293 genes that were identified as showing differential transcript abundance in the RNA-Seq data set between HP-treated and untreated conditions were examined to determine whether they belong to putative operons. Operons were defined as genes within 200 bp of one another with continuous transcript, visible as increased transcript abundance between genes, in the RNA sequencing reads using IGV. Operons were annotated and grouped into the following three categories (Table S8): (i) operons where all genes showed the same differential transcript abundance between HP-treated and untreated samples; (ii) operons where only one gene in the operon showed differential transcript abundance; and (iii) operons with a mixture, where some genes showed differential transcript abundance and some did not. In order to validate our operon annotation, RT-qPCR primers spanning the space between putative ORFs within three operons were designed. We designed qPCR primers to amplify products internal to each of the three genes, as well as primers spanning the sets of genes. Internal primer sets were compared to *omp3*, a housekeeping gene, to determine fold change and confirm differential transcript abundance. For primer pairs spanning genes, a significant cycle threshold (*C_T_*) signal value compared controls generated with no reverse transcriptase (no-RT controls) and with the housekeeping *omp3* gene, and the results were considered to show significant transcript expression differences between genes. *C_T_* values were observed for *omp3* and no-RT controls for all three operons, validating the manual operon analysis. Operon validation primers are listed in Table S1.

10.1128/mSystems.00156-18.8 TABLE S8 HP-regulated global operon annotation. Download Table S8, XLSX file, 0.04 MB. Copyright © 2018 Quillin et al. 2018 Quillin et al. This content is distributed under the terms of the Creative Commons Attribution 4.0 International license.

### Data availability.

Raw and processed sequencing results were deposited in the Gene Expression Omnibus with accession number GSE114819. The code that was used to process the sequencing results can be accessed at https://github.com/adamhockenberry/neisseria-rna-seq.
